# Highly Engineered Cr-In/H-SSZ-39 Catalyst for Enhanced Performance in CH_4_-SCR of NO*_x_*

**DOI:** 10.3390/molecules30132691

**Published:** 2025-06-21

**Authors:** Jiuhu Zhao, Jingjing Jiang, Guanyu Chen, Meng Wang, Xiaoyuan Zuo, Yanjiao Bi, Rongshu Zhu

**Affiliations:** 1 State Key Laboratory of Urban Water Resource and Environment, Shenzhen Key Laboratory of Organic Pollution Prevention and Control, School of Economics and Management, Harbin Institute of Technology, Shenzhen 518055, China; 2 School of Eco-Environment, Harbin Institute of Technology, Shenzhen 518055, China; 3 Shenzhen Government Investment Project Evaluation Center, Shenzhen 518036, China; 4 Shandong Pallet Environmental Technology Co., Ltd., Zibo 255300, China

**Keywords:** CH_4_-SCR, SSZ-39, zeolite catalyst, catalytic performance, mechanism

## Abstract

The selective catalytic reduction of NO*_x_* with CH_4_ (CH_4_-SCR) holds the potential to simultaneously abate harmful NO*_x_* and CH_4_ greenhouse gases. In this study, a series of bimetallic M-In/H-SSZ-39 catalysts (where M represents Cr, Co, Ce, and Fe) were prepared via an ion exchange method and subsequently evaluated for their CH_4_-SCR activity. The influences of the preparation parameters, including the metal ion concentration and calcination temperature, as well as the operating conditions, such as the CH_4_/NO ratio, O_2_ concentration, water vapor content, and gas hourly space velocity (GHSV), on the catalytic activity of the optimal Cr-In/H-SSZ-39 catalyst were meticulously examined. The results revealed that the Cr-In/H-SSZ-39 catalyst exhibited peak CH_4_-SCR catalytic performance when the Cr(NO_3_)_3_ concentration was 0.0075 M, the In(NO_3_)_3_ concentration was 0.066 M, and the calcination temperature was 500 °C. Under optimal operating conditions, namely GHSV of 10,000 h^−1^, 400 ppm NO, 800 ppm CH_4_, 15 vol% O_2_, and 6 vol% H_2_O, the NO*_x_* conversion rate reached 93.4%. To shed light on the excellent performance of Cr-In/H-SSZ-39 under humid conditions, a comparative analysis of the crystalline phase, chemical composition, pore structure, surface chemical state, surface acidity, and redox properties of Cr-In/H-SSZ-39 and In/H-SSZ-39 was conducted. The characterization results indicated that the incorporation of Cr into In/H-SSZ-39 enhanced its acidity and also facilitated the generation of InO^+^ active species, which promoted the oxidation of NO and the activation of CH_4_, respectively. A synergistic effect was observed between Cr and In species, which significantly improved the redox properties of the catalyst. Consequently, the activated CH_4_ could further interact with InO^+^ to produce carbon-containing intermediates such as HCOO^−^, which ultimately reacted with nitrate-based intermediates to yield N_2_, CO_2_, and H_2_O.

## 1. Introduction

Climate change and atmospheric pollution constitute pressing global challenges confronting humanity in contemporary times [1]. As a consequence, increasingly stringent emission restrictions are driving improvements in both engine technology and fuel quality around the world. Compared to diesel, liquefied natural gas (LNG) has shown significant advantages in reducing emissions such as particulate matter (PM), sulfur oxides (SO*_x_*), and carbon dioxide (CO_2_). However, LNG engines may produce higher methane (CH_4_) emissions and also have nitrogen oxide (NO*_x_*) emission issues [2,3]. CH_4_ is a greenhouse gas with high global warming potential (GWP) that is approximately 25 times (100-year horizon) greater than that of CO_2_ [4]. NO*_x_* is a prominent constituent of atmospheric pollution, posing a substantial threat to the environment and human health [5,6,7]. Therefore, the advancement of technologies focused on the simultaneous removal of NO*_x_* and CH_4_ is crucial for future progress. The selective catalytic reduction of NO*_x_* using CH_4_ (CH_4_-SCR) as a reductant holds great promise for environmental protection and energy conservation [8,9,10].

Rational catalyst design is of great significance in ensuring an efficient and durable CH_4_-SCR reaction. For a considerable period of time, efforts have been devoted to metal-exchanged medium- or large-pore zeolites characterized by their ten-membered or twelve-membered rings (MR) [9,10,11,12,13,14,15,16,17,18]. However, these catalysts either had limited catalytic activity or were susceptible to framework collapse under high-temperature and high-humidity conditions [10]. In-exchanged zeolite beta (In/H-Beta) exhibited remarkable deNO*_x_* efficiency of ~97.6% under dry CH_4_-SCR conditions. Nonetheless, once water vapor was introduced, the catalytic activity of In/H-Beta was drastically diminished [19,20]. InO^+^ species tend to combine with H_2_O, forming $In{\left(OH\right)}_{3-z}^{z+}$ species, which are unable to activate CH_4_ [18]. Moreover, prolonged exposure to H_2_O at elevated temperatures may induce the irreversible structural collapse of the zeolite framework, further compromising its catalytic performance [21]. Therefore, inhibiting the conversion of active centers and selecting a hydrothermally stable zeolite support are essential in mitigating catalyst deactivation caused by water vapor.

In the NH_3_-SCR field, the Cu-SSZ-39 zeolite (AEI topology), featuring cage-like channels with 8MR pore openings of 3.8 Å × 3.8 Å, has been highlighted for its exceptional hydrothermal stability and commendable catalytic activity [21,22,23,24,25]. Du et al. [21] conducted an in-depth investigation into the channel structure of a Cu-SSZ-39 catalyst, revealing its superior activity under humid conditions compared to its Cu-SSZ-13 counterpart. Even after undergoing high-temperature hydrothermal aging at 850 °C for 16 h, Cu-SSZ-39′s high deNO*_x_* activity was not significantly degraded. This stability was attributed to its more tortuous channel structure in comparison to SSZ-13, which effectively mitigated aluminum leaching and copper species aggregation under hydrothermal conditions. In some recent studies, SSZ-39 zeolites have also emerged as promising supports for CH_4_-SCR catalysts [26,27,28,29]. The Co-SSZ-39 catalyst synthesized by Li et al. [26] demonstrated a wider operating temperature window, higher peak NO*_x_* conversion, and more robust anti-poisoning performance compared to its Co-SSZ-13 counterpart in the CH_4_-SCR reaction. Detailed characterization of the catalyst revealed that Co^2+^ at the ion exchange sites served as the active species responsible for the catalytic activity in the CH_4_-SCR reaction. Typically, indium-exchanged zeolites are capable of catalyzing the CH_4_-SCR reaction more efficiently, making In species the preferred active components in many reported catalysts [10]. For instance, An et al. [27] reported that In/SSZ-39 exhibited superior catalytic activity compared to Cu-, Co-, and Fe/SSZ-39 catalysts. The low-temperature (400–450 °C) CH_4_-SCR activity of In/SSZ-39 was significantly enhanced with increasing indium loading within the studied range. The 3In/SSZ-39 catalyst demonstrated high NO removal efficiency of 90% and CH_4_ selectivity of 74.2% at a reaction temperature of 400 °C and a low CH_4_/NO ratio of 1. High-angle annular dark field scanning transmission electron microscopy (HAADF-STEM) images revealed that In species were highly dispersed on the SSZ-39 zeolite, which facilitated moderate CH_4_ activation and thereby enhanced the CH_4_-SCR activity at low temperatures. More importantly, the indium species loaded within the zeolite have been reported to synergize with various introduced second metals (or metal oxides), thereby significantly enhancing the activity and/or stability of the catalyst [18,28,30,31,32,33,34,35]. For instance, Chen et al. [28] constructed an In-Co_3_O_4_/H-SSZ-39(OA) catalyst through mild acid etching and ion exchange, which demonstrated improved stability under harsh operating conditions and achieved NO removal efficiency of ~80% at ~600 °C under a GHSV of 24,000 h^−1^. A small amount of Co_3_O_4_ nanoparticles on the zeolite surface greatly enhanced the catalytic activity by promoting CH_4_ conversion and enabling the greater storage of stable N*_x_*O*_y_* species at high temperatures.

In this study, a series of bimetallic M-In/H-SSZ-39 (M = Cr, Ce, Co, Fe) catalysts were prepared via a liquid-state ion exchange method, with the preparation conditions of Cr-In/H-SSZ-39—the catalyst exhibiting the highest catalytic activity—being optimized. The effects of various operating conditions on the catalytic activity of the Cr-In/H-SSZ-39 catalyst were systematically investigated. Furthermore, a comparative analysis of the crystalline phase, chemical composition, pore structure, surface chemical state, surface acidity, and redox properties of Cr-In/H-SSZ-39 and the monometallic In/H-SSZ-39 control was conducted to elucidate the underlying mechanisms responsible for the excellent catalytic activity.

## 2. Results and Discussion

### 2.1. Screening of Bimetallic Catalysts

**Figure 1:**
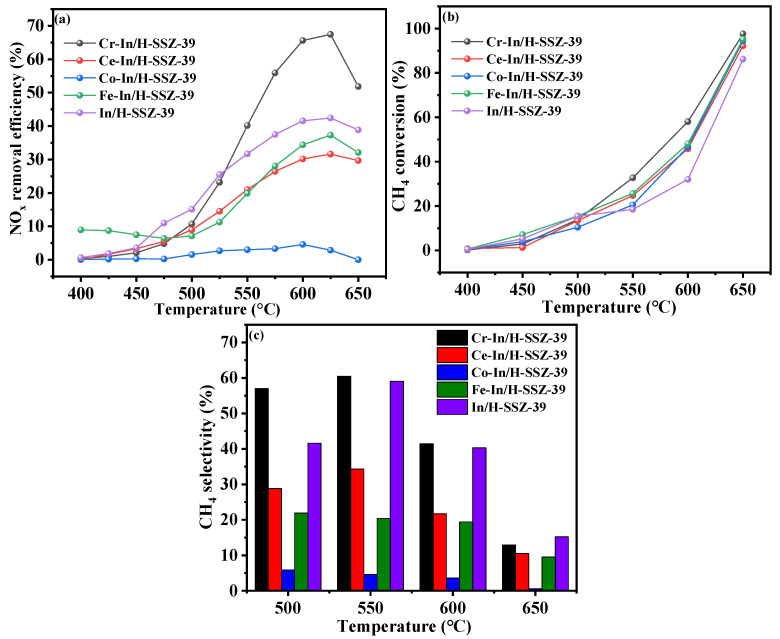
Comparison of CH_4_-SCR activity of M-In/H-SSZ-39 and In/H-SSZ-39 catalysts: (**a**) NO removal efficiency, (**b**) CH_4_ conversion, (**c**) CH_4_ selectivity. Reaction reactions: [NO] = 400 ppm, [CH_4_] = 600 ppm, [O_2_] = 10 vol%, [H_2_O] = 6 vol%, Ar balance, GHSV = 21,000 h^−1^.

Various M-In/H-SSZ-39 catalysts were prepared by introducing Cr, Ce, Co, and Fe as the second metals, and their catalytic performance was assessed, as shown in Figure 1. It is evident from Figure 1a that the incorporation of Ce, Co, and Fe species failed to effectively enhance the CH_4_-SCR activity compared with the In/H-SSZ-39 catalyst. Conversely, the incorporation of Cr species significantly enhanced the catalytic performance, achieving NO*_x_* conversion of 55% at 575 °C, further escalating to 67.8% at 620 °C. Figure 1b shows that the CH_4_ conversion of all four catalysts increased as the temperature rose. Notably, the Cr-In/H-SSZ-39 catalyst exhibited superior CH_4_ conversion at >500 °C, achieving CH_4_ conversion of 60.1% at 600 °C. The CH_4_ conversion profiles of the other three catalysts were relatively similar. Methane had two possible conversion pathways: (1) partial oxidation, which further participated in NO*_x_* reduction reactions, and (2) direct catalytic combustion. The significantly different NO*_x_* conversion and similar CH_4_ conversion rates reflected the catalysts’ varying abilities to guide methane toward the desired NO*_x_* reduction pathway, as indicated by the CH_4_ selectivity (Figure 1c). It was apparent that the incorporation of Ce, Co, and Fe as secondary metals resulted in a decrease in CH_4_ selectivity under hydrothermal conditions compared to In/H-SSZ-39. However, incorporating Cr had a beneficial effect, with Cr-In/H-SSZ-39 demonstrating the highest CH_4_ selectivity of 61.4% at 550 °C.

### 2.2. Effects of Preparation Conditions

#### 2.2.1. Effects of Cr Concentration

**Figure 2:**
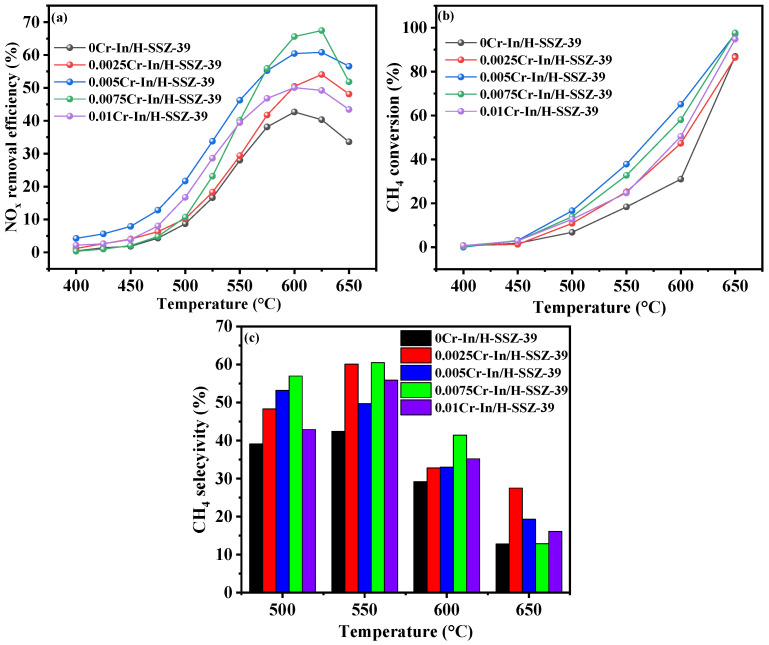
CH_4_-SCR activity of Cr-In/H-SSZ-39 catalysts prepared with different Cr(NO_3_)_3_ concentrations. (**a**) NO removal efficiency, (**b**) CH_4_ conversion, (**c**) CH_4_ selectivity. Reaction reactions: [NO] = 400 ppm, [CH_4_] = 600 ppm, [O_2_] = 10 vol%, [H_2_O] = 6 vol%, Ar balance, GHSV = 21,000 h^−1^.

Maintaining the In(NO_3_)_3_ concentration at 0.066 M, the catalytic performance of the *x*Cr-In/H-SSZ-39 (*x* = 0, 0.0025, 0.005, 0.0075, and 0.01 M Cr(NO_3_)_3_) catalysts was compared, as shown in Figure 2. With the concentration of Cr(NO_3_)_3_ increased from 0 to 0.01 M, the catalytic activity roughly exhibited an initial enhancement, followed by a gradual decline (Figure 2a). When the Cr(NO_3_)_3_ concentration was 0.0075 M, the resulting catalyst exhibited the highest NO*_x_* conversion of 67.8% at 605 °C. Figure 2b shows the CH_4_ conversion profiles of the Cr-In/H-SSZ-39 catalysts. The Cr-containing samples all exhibited enhanced CH_4_ conversion compared to the monometallic In/H-SSZ-39. Figure 2c shows that the introduction of Cr ions at varying concentrations enhanced the CH_4_ selectivity of the catalyst. Notably, the CH_4_ selectivity of 0.0075Cr-In/H-SSZ-39 was the highest at 600 °C, corresponding to its highest deNO*_x_* activity. A mixed solution of 0.0075 M Cr(NO_3_)_3_ and 0.066 M In(NO_3_)_3_ was therefore used for ion exchange in subsequent experiments.

#### 2.2.2. Effects of Calcination Temperature

**Figure 3:**
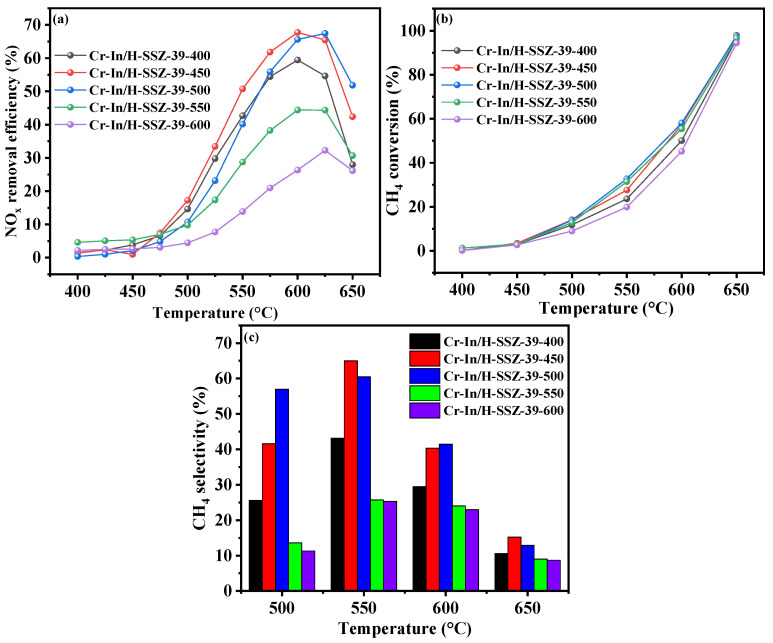
CH_4_-SCR activity of Cr-In/H-SSZ-39 catalysts calcined at different temperatures. (**a**) NO removal efficiency, (**b**) CH_4_ conversion, (**c**) CH_4_ selectivity. Reaction reactions: [NO] = 400 ppm, [CH_4_] = 600 ppm, [O_2_] = 10 vol%, [H_2_O] = 6 vol%, Ar balance, GHSV = 21,000 h^−1^.

The calcination temperature exerts a significant influence on the specific surface area of a catalyst and the chemical state of the active species [20]. To investigate the effects of the calcination temperature on the deNO*_x_* activity of the catalysts, Cr-In/H-SSZ-39 samples were calcined at different temperatures (400 °C, 450 °C, 500 °C, 550 °C, and 600 °C) and designated as Cr-In/H-SSZ-39-x, where x represents the calcination temperature. As shown in Figure 3a, the catalytic activity of the Cr-In/H-SSZ-39 catalysts initially increased and then declined with the rise in the calcination temperature. The catalysts calcined at 500 °C demonstrated the optimal CH_4_-SCR deNO*_x_* performance, high CH_4_ conversion (Figure 3b), and high CH_4_ selectivity (Figure 3c), indicating that 500 °C was the optimal calcination temperature for the Cr-In/H-SSZ-39 catalyst.

### 2.3. Effects of Reaction Conditions

#### 2.3.1. CH_4_/NO Ratio

**Figure 4:**
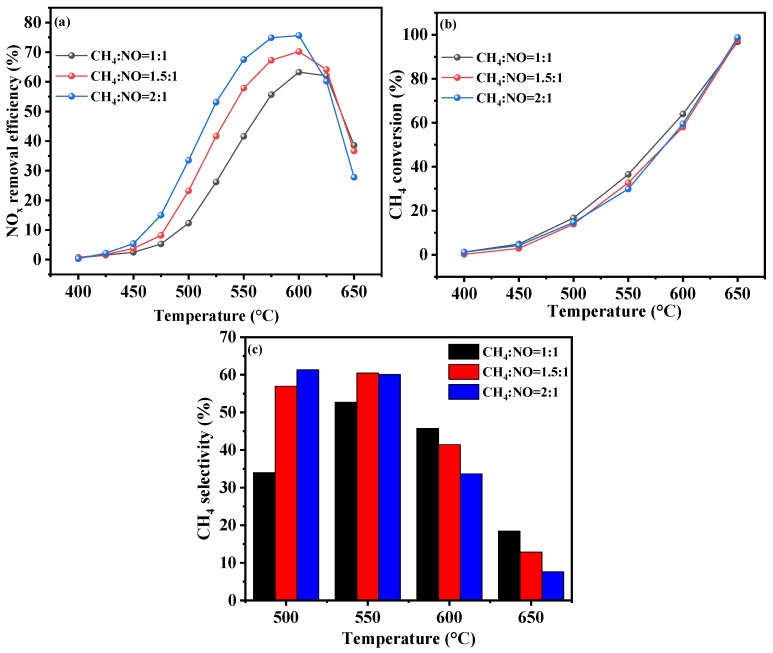
CH_4_-SCR activity of Cr-In/H-SSZ-39 catalysts under different CH_4_/NO ratios. (**a**) NO removal efficiency, (**b**) CH_4_ conversion, and (**c**) CH_4_ selectivity. Reaction conditions: [NO] = 400 ppm, [CH_4_] = 400/600/800 ppm, [O_2_] = 10 vol%, [H_2_O] = 6 vol%, Ar balance, GHSV = 21,000 h^−1^.

The CH_4_-SCR process removes NO through the reaction of methane, nitric oxide, and oxygen, generating harmless nitrogen, water, and low-GWP carbon dioxide. The CH_4_/NO ratio is a key indicator in assessing the effectiveness of the reaction. Figure 4a illustrates the catalytic activity of the Cr-In/H-SSZ-39 catalysts under various CH_4_/NO ratios. At a CH_4_/NO ratio of 1.0, Cr-In/H-SSZ-39 exhibited excellent catalytic activity in the high-temperature range, achieving a peak NO*_x_* conversion of 59.6% at 600 °C. By progressively elevating the CH_4_ concentration in the feed gas, the catalyst’s activity at <600 °C was continuously enhanced. A reasonable explanation is that the higher CH_4_ initial concentration at a fixed NO*_x_* concentration allowed more CH_4_ to be involved in NO*_x_* reduction at low temperatures, where CH_4_ activation was relatively difficult. At a CH_4_/NO ratio of 1.5, the Cr-In/H-SSZ-39 catalyst demonstrated its peak NO*_x_* conversion of 67.8% at 600 °C. When the CH_4_/NO ratio was further increased to 2, the catalyst’s peak NO*_x_* conversion reached 76.1% at 600 °C. However, an excessively high concentration of CH_4_ could not be fully converted over the catalyst, leading to the direct emission of high-GWP CH_4_. It is evident from Figure 4b that the CH_4_ conversion decreased as the CH_4_/NO ratio increased. Moreover, as shown in Figure 4c, a decrease in the CH_4_ selectivity at >600 °C was observed as the CH_4_/NO ratio increased, which might be attributed to the fact that a major portion of CH_4_ was catalytically combusted at high temperatures. At CH_4_/NO ratios of 1.5 and 2, the CH_4_ selectivity at 500 and 550 °C was similar, with a higher supply of CH_4_ naturally leading to enhanced deNO*_x_* activity.

#### 2.3.2. O_2_ Concentration

**Figure 5:**
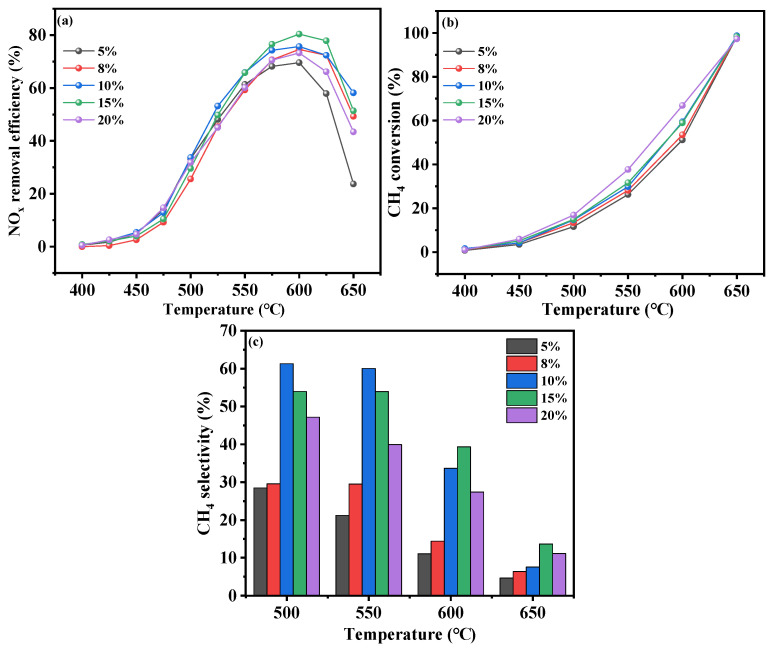
CH_4_-SCR activity of Cr-In/H-SSZ-39 catalysts under different O_2_ concentrations. (**a**) NO removal efficiency, (**b**) CH_4_ conversion, and (**c**) CH_4_ selectivity. Reaction conditions: [NO] = 400 ppm, [CH_4_] = 600 ppm, [O_2_] = 5/8/10/15/20 vol%, [H_2_O] = 6 vol%, Ar balance, GHSV = 21,000 h^−1^.

In CH_4_-SCR, inadequate O_2_ concentrations might result in incomplete reactions between CH_4_ and NO*_x_*, whereas excessive ones might prompt the overcombustion of CH_4_. Figure 5a depicts the NO*_x_* conversion profile of the Cr-In/H-SSZ-39 catalyst under different O_2_ concentrations. The catalyst exhibited optimal deNO*_x_* performance at an O_2_ concentration of 10 vol%, with stable catalytic performance exceeding 60% above 550 °C. Specifically, at 600 °C, the deNO*_x_* performance remained stable above 60%, achieving peak catalytic performance of 80.6%. However, as the O_2_ concentration was further increased to 20 vol%, the catalytic performance of the catalyst declined. As observed in Figure 5b, CH_4_ conversion increased with the rise in the O_2_ concentration, primarily due to the enhanced oxidation kinetics of CH_4_ under oxygen-rich conditions. Specifically, the highest CH_4_ conversion occurred at 20 vol% O_2_, followed by comparable CH_4_ conversion at 15 vol% and 10 vol% O_2_. As shown in Figure 5c, the CH_4_ selectivity exhibited a trend similar to that of the NO*_x_* removal efficiency with respect to the O_2_ concentration. This correlation can be attributed to the fact that, at an O_2_ concentration of 20 vol%, CH_4_ more readily reacts with oxygen, thereby reducing its selectivity toward NO*_x_*.

#### 2.3.3. Gaseous Hourly Space Velocity

**Figure 6:**
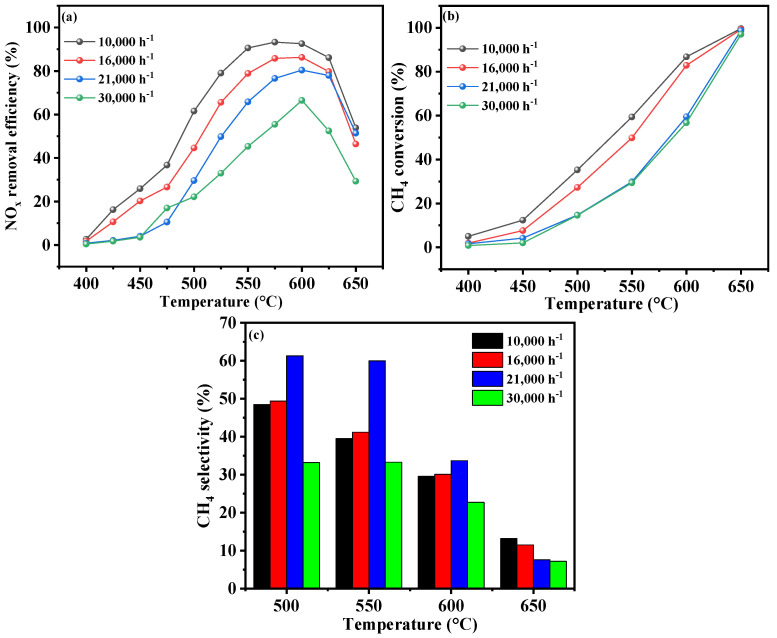
CH_4_-SCR activity of Cr-In/H-SSZ-39 catalyst under different GHSVs. (**a**) NO*_x_* removal efficiency, (**b**) CH_4_ conversion, and (**c**) CH_4_ selectivity. Reaction conditions: [NO] = 400 ppm, [CH_4_] = 600 ppm, [O_2_] = 10 vol%, [H_2_O] = 6 vol%, Ar balance, GHSV = 10,000/16,000/21,000/30,000 h^−1^.

The influence of the GHSV on the deNO*_x_* performance of the Cr-In/H-SSZ-39 catalyst was also investigated, as shown in Figure 6. It could be found that the NO*_x_* conversion gradually increased with the reduction in GHSV from 30,000 h^−1^ to 10,000 h^−1^ (Figure 6a). At a GHSV of 16,000 h^−1^, the catalyst achieved the peak catalytic activity at 600 °C, with an NO*_x_* conversion rate of 86.1%. When the GHSV was further decreased to 10,000 h^−1^, the catalyst demonstrated its highest deNO*_x_* activity at a slightly lower temperature of 590 °C, with remarkable NO*_x_* conversion of 93.4%. A decreasing GHSV led to an increase in CH_4_ conversion (Figure 6b). CH_4_ was more effectively reacted within the temperature range of 400–650 °C at a lower GHSV. Furthermore, Figure 6c reveals that the CH_4_ selectivity remained high at a GHSV of 10,000 h^−1^, indicating that CH_4_ predominantly participated in NO*_x_* reduction.

#### 2.3.4. Water Vapor Content

**Figure 7:**
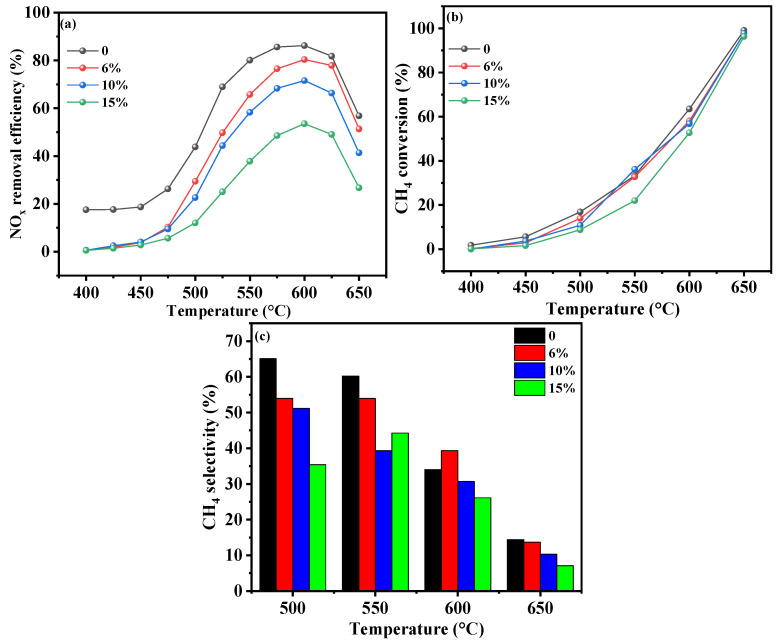
CH_4_-SCR activity of Cr-In/H-SSZ-39 catalyst under different H_2_O concentrations. (**a**) NO*_x_* removal efficiency, (**b**) CH_4_ conversion, and (**c**) CH_4_ selectivity. Reaction conditions: [NO] = 400 ppm, [CH_4_] = 600 ppm, [O_2_] = 10 vol%, [H_2_O] = 0/6/10/15 vol%, Ar balance, GHSV = 21,000 h^−1^.

The tolerance of the Cr-In/H-SSZ-39 catalyst to water vapor was examined (Figure 7). In the absence of water vapor, the catalyst achieved high NO*_x_* conversion of 86% at 580 °C (Figure 7a). Peak NO*_x_* conversion of 80.6% was observed at 600 °C when 6 vol% water vapor was introduced. As the water content was further increased to 10 vol% and 15 vol%, the peak NO*_x_* conversion declined to 70.6% and 52%, respectively. These findings underscore the robust hydrothermal stability of the Cr-In/H-SSZ-39 catalyst. Figure 7b shows that CH_4_ conversion declined with increasing water content, indicating that water vapor inhibited CH_4_ conversion. At water vapor content of 10 vol%, the CH_4_ conversion of the catalyst was 51.3% at 600 °C, a value between those observed at 6 vol% and 15 vol% water vapor content. Figure 7c shows the direct correlation between the CH_4_ selectivity and catalytic performance at temperatures below 550 °C. When the water vapor content was 6 vol%, the CH_4_ selectivity at 600 °C exceeded that observed without water vapor, indicating that water vapor might exert a stronger inhibitory effect on CH_4_ combustion.

### 2.4. Cyclic and Stability Testing

**Figure 8:**
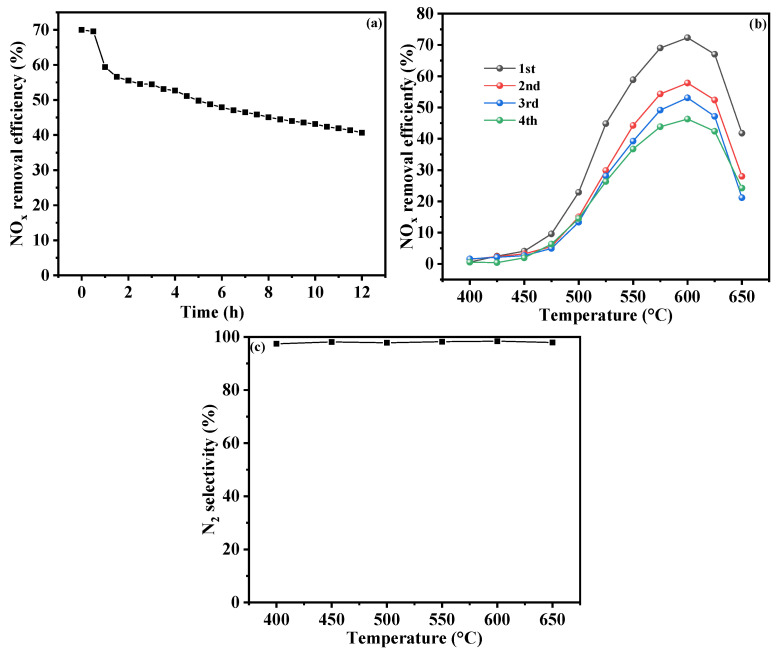
(**a**) Long-term operation test for Cr-In/H-SSZ-39, (**b**) catalytic activity of Cr-In/H-SSZ-39 in three consecutive TPSR cycles, and (**c**) N_2_ selectivity test. Reaction conditions: [NO] = 400 ppm, [CH_4_] = 800 ppm, [O_2_] = 15 vol%, [H_2_O] = 10 vol%, Ar balance, GHSV = 21,000 h^−1^.

The results of the long-term operation test and three consecutive TPSR tests of the Cr-In/H-SSZ-39 catalyst are presented in Figure 8. At a constant temperature of 600 °C, the NO*_x_* conversion of the catalyst decreased from 70% to 59% after 1 h of exposure to hydrothermal conditions (Figure 8a). Following this initial decline, the conversion exhibited a gradual and steady decrease over time. In the presence of 10 vol% H_2_O, the catalytic activity remained above 50% for up to 5 h and above 40% for up to 12 h. These results demonstrate the robust stability of the Cr-In/H-SSZ-39 catalyst under high-temperature hydrothermal conditions. As shown in Figure 8b, the Cr-In/H-SSZ-39 catalyst retained a high level of catalytic activity, achieving NO removal efficiency of 58% and 51% in the second and third cycles, respectively. Compared with the In/H-Beta catalyst [20], the Cr-In/H-SSZ-39 catalyst exhibited higher stability after multiple cycles. The N_2_ selectivity, as an essential indictor, was assessed, and the results are presented in Figure 8c. It can be seen that the N_2_ selectivity exceeds 99%, with almost no N_2_O being produced.

### 2.5. Characterization and Analysis of the Cr-In/H-SSZ-39 Catalyst

#### 2.5.1. Analysis of Composition and Texture Properties

**Figure 9:**
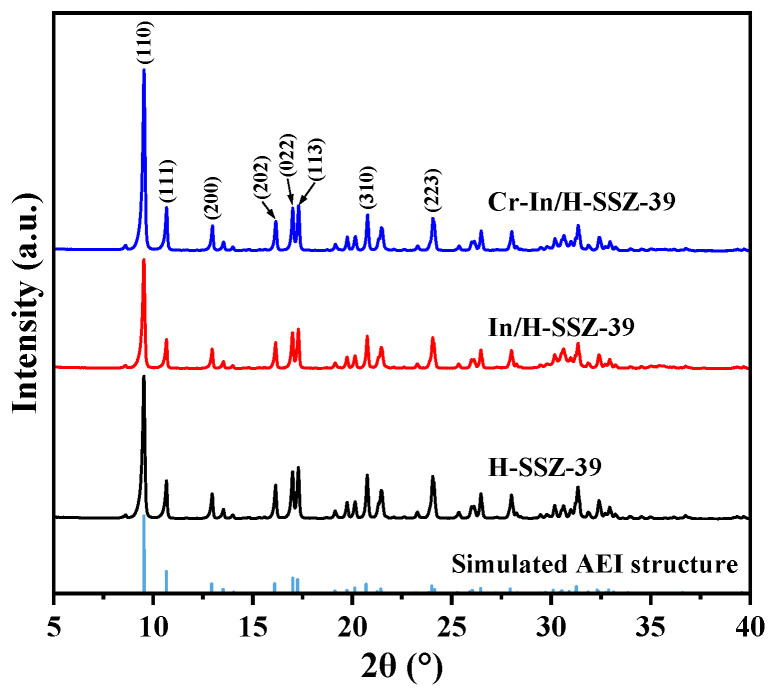
PXRD patterns of H-SSZ-39, In/H-SSZ-39, and Cr-In/H-SSZ-39. The data of the simulated AEI structure are from the Database of Zeolite Structures.

**Figure 10:**
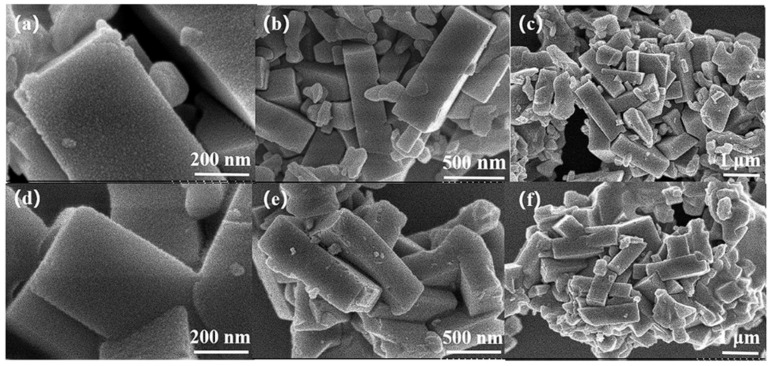
SEM images of (**a**–**c**) In/H-SSZ-39 and (**d**–**f**) Cr-In/H-SSZ-39 catalysts.

**Figure 11:**
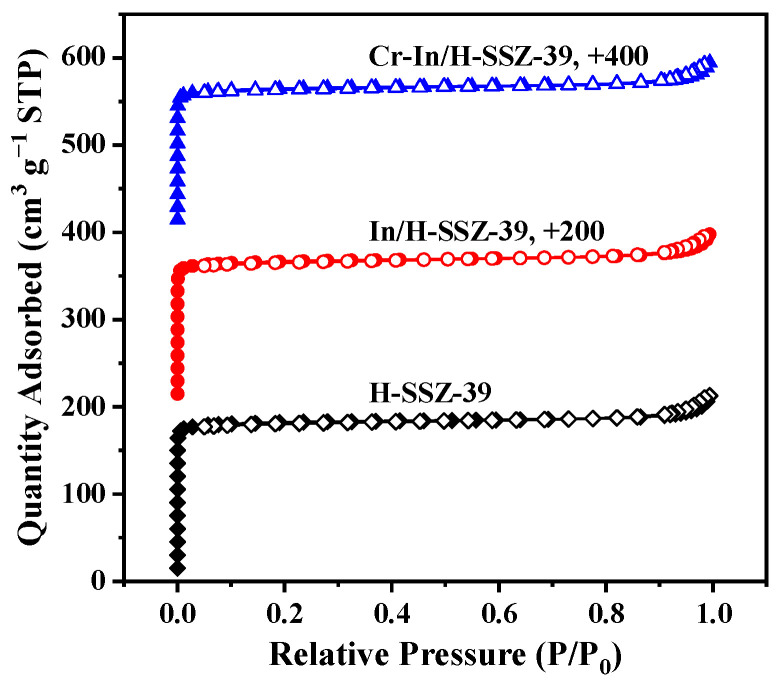
N_2_ adsorption–desorption isotherms of H-SSZ-39, In/H-SSZ-39, and Cr-In/H-SSZ-39 samples.

Figure 9 shows the PXRD patterns of the H-SSZ-39, In/H-SSZ-39, and Cr-In/H-SSZ-39 samples. The characteristic diffraction peaks (9.5°, 10.7°, 13.0°, 16.2°, 17.0°, 17.3°, 20.8°, and 24.1°) of each sample aligned well with the simulated AEI structure, indicating that the SSZ-39 zeolite remained intact after incorporating In and Cr species. For zeolite-based deNO*_x_* catalysts, maintaining a stable crystalline structure is crucial to ensure the distribution of metal species in their exchangeable sites. The micromorphologies of the In/H-SSZ-39 and Cr-In/H-SSZ-39 catalysts were observed by SEM, as shown in Figure 10. There was no discernible difference in the micromorphologies of the two samples, both consisting of aggregated cuboid crystals with smooth surfaces. The surface area and pore structure of the catalyst influence the dispersion and accessibility of the active species. As shown in Figure 11 and Table 1, the Cr-In/H-SSZ-39 and In/H-SSZ-39 catalysts presented micropore-dominant pore structure, evidenced by the type I N_2_ adsorption–desorption isotherms (Figure 11) and steep N_2_ adsorption behavior at *P*/*P*_0_ < 0.01. The preserved intrinsic microporosity characteristics demonstrated that the introduction of Cr/In species did not cause significant disruption to the ordered framework structure of the SSZ-39 zeolite, in line with the PXRD patterns and SEM observations.

To determine the elemental compositions of the catalysts, the ICP-OES technique was employed, as summarized in Table 2. The Cr content in Cr-In/H-SSZ-39 was 0.075 wt%, indicating that the Cr ions (0.0075 M) in the ion exchange solution migrated into the zeolites. In/H-SSZ-39 exhibited In content of 5.5 wt%, whereas the In content of Cr-In/H-SSZ-39 slightly decreased to 4.7 wt%, suggesting that In and Cr ions might compete for zeolites’ finite exchangeable sites during the ion exchange process.

#### 2.5.2. Analysis of Chemical States and Redox Properties

**Figure 12:**
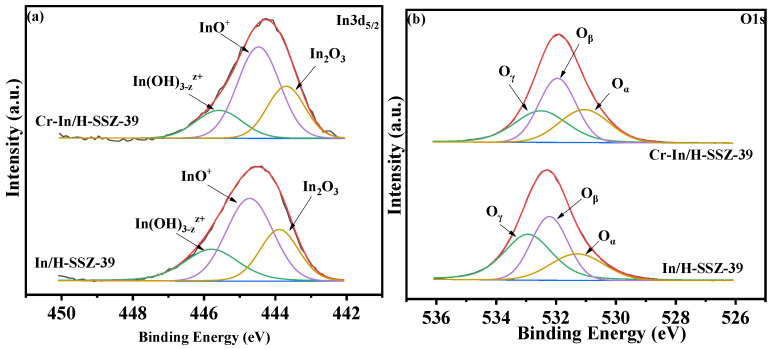
XPS spectra of (**a**) In 3d_5/2_ and (**b**) O 1s spectra of In/H-SSZ-39 and Cr-In/H-SSZ-39 catalysts.

Figure 12a presents the In 3d_5/2_ XPS spectra of the In/H-SSZ-39 and Cr-In/H-SSZ-39 catalysts, and the according calculations are summarized in Table 3. Spectral deconvolution allowed for the identification of three indium species: $In{\left(OH\right)}_{3-z}^{z+}$ (~445.6 eV), InO^+^ (~444.5 eV), and In_2_O_3_ (~443.9 eV) [29]. Furthermore, a discernible shift in the In 3d_5/2_ spectra toward higher binding energies was observed for Cr-In/H-SSZ-39 compared to In/H-SSZ-39, which could be explained by the interaction between Cr and In species. Higher binding energy is indicative of more oxidative surface chemistry, which promotes stronger interactions with SCR reactant species and, consequently, enhances NO*_x_* conversion. Table 3 shows that the Cr-In/H-SSZ-39 catalyst demonstrated a larger proportion of InO^+^ species (InO^+^/($In{\left(OH\right)}_{3-z}^{z+}$ + InO^+^ + In_2_O_3_) = 0.54) compared to In/H-SSZ-39 (0.49). Given that InO^+^ is widely recognized as the primary active site in In-exchanged zeolite CH_4_-SCR catalysts [18,30,33,34], more InO^+^ species might also contribute to the exceptional deNO*_x_* activity of Cr-In/H-SSZ-39. Figure 12b shows the O 1s XPS spectra of the two catalysts, which were deconvoluted into three distinct peaks, i.e., oxygen adsorbed on hydroxyl groups (O_γ_, 532.4–533.1 eV), surface oxygen species (O_β_, 532.1–532.6 eV), and lattice oxygen (O_α_, 531.0–531.5 eV) [20,36,37,38]. Notably, the O_β_ content of Cr-In/H-SSZ-39 reached 40.4%, representing a 3.9% increase compared to that of In/H-SSZ-39 (36.5%). Introducing Cr species into the In/H-SSZ-39 catalyst led to an increase in the O_β_ content. Considering that surface oxygen is more involved in complete oxidation reactions, the higher surface oxygen content of Cr-In/H-SSZ-39 promotes CH_4_ activation [19], thereby enhancing its catalytic activity.

**Figure 13:**
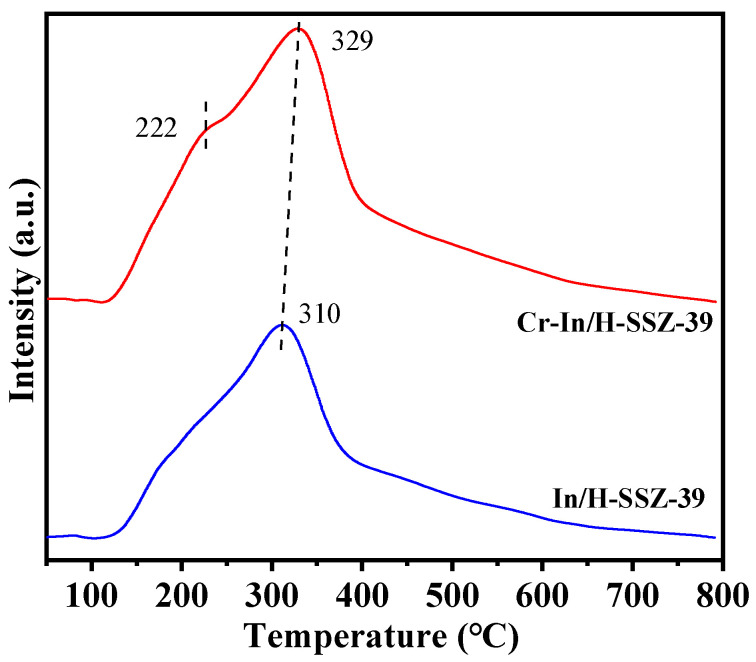
H_2_-TPR profiles of In/H-SSZ-39 and Cr-In/H-SSZ-39 catalysts.

The redox properties of the two catalysts were investigated by the H_2_-TPR method, as depicted in Figure 13. Cr-In/H-SSZ-39 exhibited a reduction peak at 222 °C, attributable to the reduction of Cr^6+^ to Cr^3+^ [39]. Additionally, a prominent reduction peak was observed at 310 °C/329 °C, which corresponded to the reduction of In^3+^ [30]. Notably, the reduction peak of InO^+^ in the Cr-In/H-SSZ-39 catalyst shifted to a higher temperature in comparison to that of the In/H-SSZ-39 catalyst. This finding suggests a synergistic interaction between Cr and In species [40].

**Figure 14:**
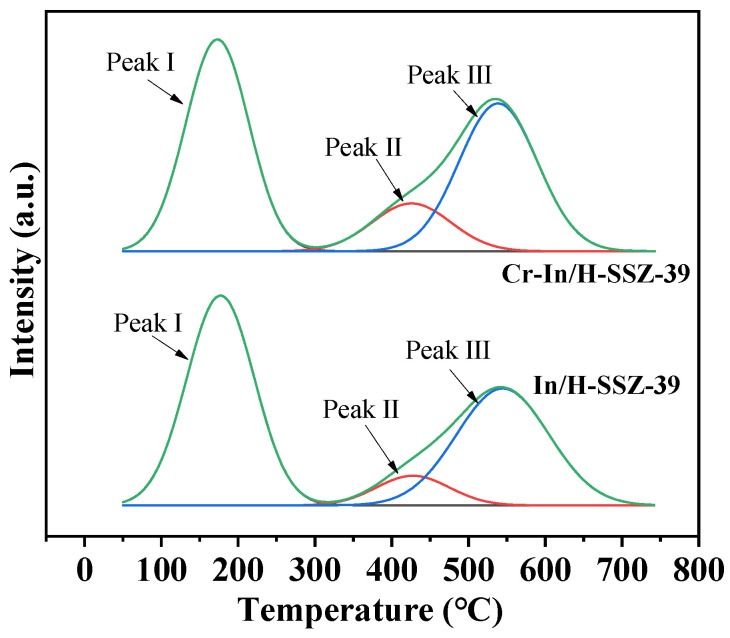
NH_3_-TPD profiles of In/H-SSZ-39 and Cr-In/H-SSZ-39 catalysts.

The acidity of the two catalysts was investigated by NH_3_-TPD, and the results are shown in Figure 14 and Table 4. The NH_3_-TPD profiles were deconvoluted into three distinct peaks: peak I at ~180 °C, attributed to surface hydroxyl groups and weak Lewis acid sites (LAS); peak II at ~470 °C, associated with strong LAS; and peak III at ~540 °C, linked to strong Brønsted acids. Notably, Cr-In/H-SSZ-39 demonstrated 11.3% higher total acidity (2.018 mmol g^−1^ vs. 1.813 mmol g^−1^) than its In/H-SSZ-39 counterpart. This observation aligns well with the trend observed in the CH_4_-SCR performance, thereby reinforcing the pivotal role of acidic sites in enhancing catalytic activity [29].

### 2.6. Characterization of the Cr-In/H-SSZ-39 Catalyst After Reaction

#### 2.6.1. Analysis of Chemical States

**Figure 15:**
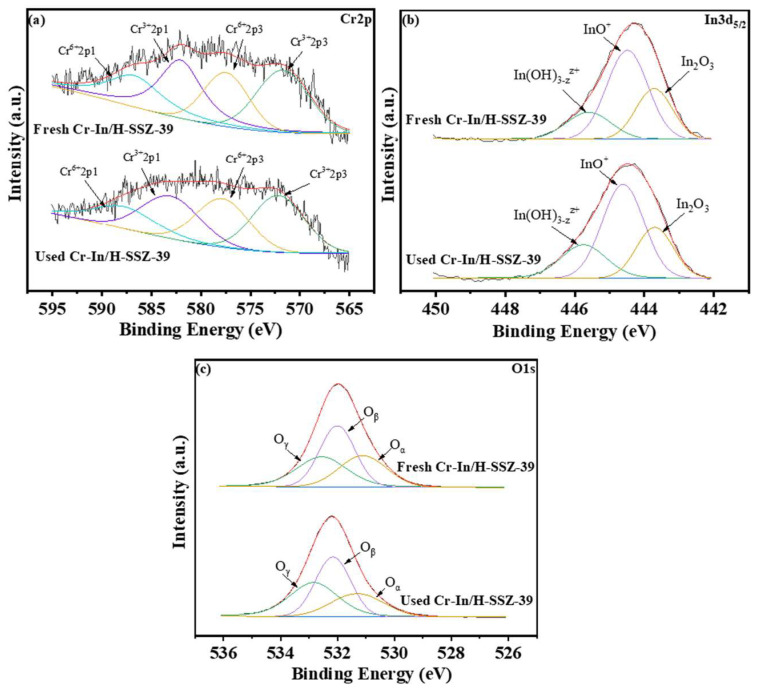
XPS (**a**) Cr 2p, (**b**) In 3d_5/2_, and (**c**) O 1s spectra of the fresh and used Cr-In/H-SSZ-39 catalysts.

Figure 15a and Table 4 present a comparison of the Cr 2p spectra for the Cr-In/H-SSZ-39 catalyst before and after the reaction. XPS spectral deconvolution resolved characteristic Cr^3+^ (2p_3/2_ at 571.8 eV, 2p_1/2_ at 588.1 eV) and Cr^6+^ (2p_3/2_ at 577.5 eV, 2p_1/2_ at 586.8 eV) oxidation states. Cr^3+^ played a pivotal role in generating oxygen vacancies on the catalyst surface. Oxygen vacancies serve as active sites that promote reactant activation via interacting with NO_2_ to generate surface-adsorbed nitrite ($NO_{2}^{-}$) and nitrate ($NO_{3}^{-}$) intermediates, ultimately contributing to the enhanced catalytic performance [41]. The Cr^3+^/Cr^6+^ ratios of the fresh and used Cr-In/H-SSZ-39 catalysts were 0.58 and 0.57, respectively, indicating that the catalyst retained a considerable number of Cr^3+^ active sites, which were essential for its effective catalytic performance. Figure 15b shows the In 3d_5/2_ spectra of the fresh and used Cr-In/H-SSZ-39 catalysts, which could be deconvoluted into three distinct indium species: $In{\left(OH\right)}_{3-z}^{z+}$, InO^+^, and In_2_O_3_. The calculated InO^+^/(InO^+^ + In_2_O_3_ + $In{\left(OH\right)}_{3-z}^{z+}$) ratio of the used Cr-In/H-SSZ-39 catalyst remained stable at 0.53, almost identical to the value of the fresh one (0.54). Additionally, as shown in Figure 15c, the content of O_β_ was almost unchanged after the reaction under humid conditions, decreasing slightly from 40.4% to 40.2%. The consistent Cr^3+^/Cr^6+^, InO^+^/(InO^+^ + In_2_O_3_ + $In{\left(OH\right)}_{3-z}^{z+}$), and O_β_ ratios explain the excellent cyclability and hydrothermal stability of the Cr-In/H-SSZ-39 catalyst.

#### 2.6.2. Analysis of Acidity

**Figure 16:**
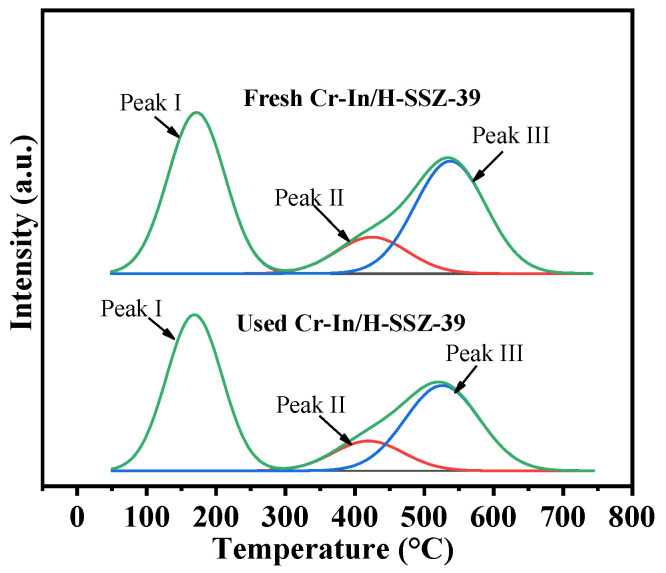
NH_3_-TPD profiles of fresh and used Cr-In/H-SSZ-39 catalysts.

Figure 16 shows the NH_3_-TPD profiles of the fresh and used Cr-In/H-SSZ-39 catalysts, which could be deconvoluted into three NH_3_ desorption peaks. The NH_3_ desorption from the used Cr-In/H-SSZ-39 catalyst at the three temperature ranges was slightly lower than that of the fresh catalyst, indicating the reduced acidity of the catalyst after the reaction (Table 4). The minor loss of acidity after the reaction might also have contributed to the retention of its strong deNO*_x_* performance.

### 2.7. Analysis of Water Resistance Mechanism

Based on our previous study of the CH_4_-SCR deNO*_x_* mechanism over In/H-Beta [38], BAS could induce a small amount of In_2_O_3_ on the zeolite surface to generate InO^+^ active sites; CH_4_ is mainly adsorbed on InO^+^ sites, which provide the activated oxygen (O*) necessary for CH_4_ activation. Combining this with the present research results, the CH_4_-SCR deNO*_x_* mechanism over the Cr-In/H-SSZ-39 catalyst is as follows:

1. It could be seen from PXRD that the catalyst maintained its crystalline properties to a great extent after introducing Cr and In species;
2. The NH_3_-TPD and XPS results showed that the incorporation of Cr into In/H-SSZ-39 increased the number of BAS and generated more InO^+^ species, thus promoting NO oxidation and CH_4_ activation;
3. A strong interaction between Cr and In species was found from the XPS and H_2_-TPR results; introducing Cr species increased the redox properties of the catalyst, thus promoting NO oxidation.

## 3. Experimental Section

### 3.1. Catalyst Preparation

#### 3.1.1. Chemicals

H-SSZ-39 zeolite with an Si/Al ratio of 16 was provided by China Catalyst Holding Co., Ltd. (Zibo, China). Indium(III) nitrate hydrate (In(NO_3_)_3_·*x*H_2_O, 99.9% metal basis, indium content of 28–37%) was acquired from Shanghai Aladdin Biochemical Technology Co., Ltd. (Shanghai, China). Chromium(III) nitrate nonahydrate (Cr(NO_3_)_3_·9H_2_O, 99.95% metal basis), cerium(III) nitrate hexahydrate (Ce(NO_3_)_3_·6H_2_O, 99.5% metal basis), cobalt(II) nitrate hexahydrate (Co(NO_3_)_2_·6H_2_O, 99.99% metal basis), and iron(III) nitrate nonahydrate (Fe(NO_3_)_3_·9H_2_O, 99.9% metal basis) were bought from Shanghai Macklin Biochemical Technology Co., Ltd. (Shanghai, China). All reagents were used as received, without further purification. Home-made ultrapure water (18.25 MΩ·cm) was used throughout the entire experiment.

#### 3.1.2. Preparation of Monometallic In/H-SSZ-39 Catalyst

The In/H-SSZ-39 catalyst was prepared via a liquid-state ion exchange method. First, 100 mL of 0.066 M In(NO_3_)_3_ aqueous solution was prepared by dissolving In(NO_3_)_3_·*x*H_2_O in ultrapure water. Subsequently, 3 g of H-SSZ-39 zeolite was dispersed into the In(NO_3_)_3_ solution. The resultant mixture was uniformly agitated at a constant temperature of 85 °C for 8 h using a magnetic stirrer. The solid was recovered by centrifugation, thoroughly washed five times with deionized water, and then dried in an oven at 80 °C for 12 h. Lastly, the dried sample was calcined in a muffle furnace at 500 °C for 3 h, after which it was sealed and stored for future use.

#### 3.1.3. Preparation of Bimetallic M-In/H-SSZ-39 Catalyst

The preparation process of the M-In/H-SSZ-39 catalyst was similar to that of In/H-SSZ-39, except that a mixed aqueous solution of In(NO_3_)_3_ and the second metal M precursor (Cr(NO_3_)_3_·9H_2_O, Ce(NO_3_)_3_·6H_2_O, Co(NO_3_)_2_·6H_2_O, and Fe(NO_3_)_3_·9H_2_O) was used. Variations were introduced into the synthesis process to optimize the properties of the Cr-In/H-SSZ-39 catalyst. For Cr content optimization, different amounts of Cr(NO_3_)_3_·9H_2_O (0.11 g, 0.21 g, 0.32 g, and 0.43 g) were dissolved in 100 mL of 0.066 M In(NO_3_)_3_ aqueous solution during the initial step. To determine the optimal calcination temperature, the materials were calcined at 450 °C, 500 °C, 550 °C, and 600 °C in the final step.

### 3.2. Catalytic Activity Measurement

The activity evaluation experiments were conducted at atmospheric pressure using the temperature-programmed surface reaction (TPSR) method. A certain mass of catalyst particles (40–60 mesh) was first loaded in a fixed-bed quartz reactor. The reaction gas mixture composed of 400 ppm NO, 600 ppm CH_4_, 10 vol% O_2_, and 6 vol% H_2_O with an Ar balance at a total flow rate of 100 mL min^−1^ was introduced into the reactor, corresponding to a GHSV of ~21,000 h^−1^ for 0.1 g of catalyst particles. After establishing NO*_x_* adsorption–desorption equilibrium at 100 °C, a programmed temperature ramp was initiated, gradually raising the reactor temperature from 100 °C to 675 °C at a heating rate of 5 °C min^−1^. The concentrations of NO*_x_* were monitored by a nitrogen oxide analyzer (Teledyne Model T200H, Teledyne Monitor Labs, Inc., Centennial, CO, USA), while the CH_4_, CO, and CO_2_ concentrations were analyzed by a gas chromatograph (Fuli GC9790II, China) equipped with a Porapak-Q column (Agilent, Santa Clara, CA, USA) and a flame ionization detector (FID). A gas chromatograph (Agilent 7890B) was used to detect the N_2_ content; the detectors were TCD and ECD detectors; a 5A molecular sieve column was used; and the carrier gases were N_2_ and He. The CH_4_-SCR activity of the catalyst was assessed using the following parameters: NO*_x_* removal efficiency (η), CH_4_ conversion (γ), CH_4_ selectivity (α), and N_2_ selectivity (S_N2_). These were calculated using Equations (1), (2), (3), and (4), respectively:

(1) $$\eta =\frac{c{\left({NO}_{x}\right)}_{\mathrm{in}}-c{\left({NO}_{x}\right)}_{out}}{c{\left({NO}_{x}\right)}_{in}}\times 100\%$$

(2) $$\gamma =\frac{c{\left({CH}_{4}\right)}_{in}-c{\left({CH}_{4}\right)}_{out}}{c{\left({CH}_{4}\right)}_{in}}\times 100\%$$

(3) $$\alpha =\frac{0.5(c{\left({NO}_{x}\right)}_{\mathrm{in}}-c{\left({NO}_{x}\right)}_{out})}{c{\left({CH}_{4}\right)}_{in}-c{\left({CH}_{4}\right)}_{out}}\times 100\%$$

(4) $$S_{N2}=\frac{{2c(N_{2})}_{out}}{c{\left({NO}_{x}\right)}_{in}-c{\left({NO}_{x}\right)}_{\mathrm{out}}}\times 100\%$$

where c(NO_x_)_in_ is the input concentration of NO_x_, c(NO_x_)_out_ is the output concentration of NO_x_, c(CH_4_)_in_ is the input concentration of CH_4_, c(CH_4_)_out_ is the output concentration of CH_4_, and c(N_2_)_out_ is the concentration of N_2_ formed, respectively.

### 3.3. Catalyst Characterization

Scanning electron microscopy (SEM) observation was conducted using a Thermo Scientific Apreo 2S HiVac (Waltham, MA, USA). To understand the crystalline phases and structures of the catalysts, powder X-ray diffraction (PXRD) patterns were recorded by a Bruker D8 Venture diffractometer (Germany) in the diffraction angle range of 2*θ* = 5–40° (2° min^−1^) with Cu K*α* radiation (*λ* = 1.5418 Å) at 40 kV and 40 mA. Inductively coupled plasma optical emission spectrometry (ICP-OES, Agilent 720ES, USA) was used to determine the elemental content of Cr, In, Si, and Al in the bulk catalyst. The specific surface area and pore structure of the catalyst samples were calculated from N_2_ adsorption–desorption isotherms measured at 77 K on a Micromeritics ASAP 2460 instrument (Micromeritics, GA, USA). Prior to measurement, the samples were degassed at 300 °C for 6 h to eliminate adsorbed impurities. The surface elemental composition and chemical state of the catalyst were analyzed using X-ray photoelectron spectroscopy (XPS) on an ESCALAB MK-II electron spectrometer (Thermo Scientific, Waltham, MA, USA) with a monochromatic Al K*α* radiation source (1486.6 eV). The binding energy values were calibrated using the C 1s peak at 284.8 eV for adventitious carbon. The reducibility of the catalysts was evaluated by temperature-programmed reduction with hydrogen (H_2_-TPR) using an AutoChem Ⅱ 2920 instrument (Micromeritics, Norcross, GA, USA). Before the measurement, 50 mg samples (40–60 mesh) were loaded into a U-shaped quartz tube and pretreated at 500 °C in an He gas flow of 30 mL/min for 2 h. After being cooled to 50 °C under the same atmosphere, the samples were exposed to a 10 vol% H_2_/Ar mixture at a flow rate of 30 mL/min and heated from 50 °C to 650 °C at a ramp rate of 10 °C/min. Temperature-programmed desorption of ammonia (NH_3_-TPD) was conducted using a 5 vol% NH_3_/N_2_ gas mixture under a similar temperature program to investigate the acidic properties of the samples.

## 4. Conclusions

In summary, a Cr-In/H-SSZ-39 catalyst with high CH_4_-SCR deNO*_x_* performance was successfully prepared, and its preparation conditions were systematically optimized. The effects of the operating conditions were investigated, and the CH_4_-SCR deNO*_x_* mechanism over Cr-In/H-SSZ-39 was elucidated. The main findings of this study are as follows:

(1) The Cr-In/H-SSZ-39 catalyst exhibited optimal NO*_x_* conversion of 80% when exchanged with a mixed solution of 0.0075 M Cr(NO_3_)_3_ and 0.066 M In(NO_3_)_3_, calcined at 500 °C, and tested under conditions of 400 ppm NO, 600 ppm CH_4_, 10 vol% O_2_, 6 vol% H_2_O, and a GHSV of 21,000 h^−1^. The catalytic activity of Cr-In/H-SSZ-39 under humid conditions was significantly superior to that of the monometallic In/H-SSZ-39.

(2) Under the optimized operating conditions, the Cr-In/H-SSZ-39 catalyst demonstrated excellent regeneration ability and long-term stability. Specifically, under 10 vol% H_2_O content and at 600 °C, the NO*_x_* conversion in four consecutive cycles were 71%, 58%, 51%, and 43%, respectively. The long-term operation test further demonstrated that the NO*_x_* removal efficiency was kept at over 50% for up to 5 h and above 40% for up to 12 h.

(3) The incorporation of Cr into the In/H-SSZ-39 catalyst resulted in an increase in BAS and the production of more InO^+^ active species, which were responsible to the oxidation of NO and the activation of CH_4_. A potent synergistic interaction was observed between Cr and In species. The additional Cr^3+^ species significantly enhanced the redox properties of the catalyst, further promoting the oxidation of NO and the activation of CH_4_, ultimately leading to an improvement in catalytic activity. This approach holds promise as a feasible strategy to enhance the catalytic performance of In/H-SSZ-39 under harsh high-temperature hydrothermal conditions commonly encountered in practical applications.

## Figures and Tables

**Table 1:** Textural properties of In/H-SSZ-39 and Cr-In/H-SSZ-39 samples.

Sample	*S*_BET_ ^a^	*V*_total_ ^b^	*V*_micro_ ^c^
	(m^2^ g^−1^)	(cm^3^ g^−1^)	(cm^3^ g^−1^)
H-SSZ-39	754.5	0.293	0.269
In/H-SSZ-39	685.5	0.271	0.243
Cr-In/H-SSZ-39	676.7	0.267	0.240

^a^ BET surface area obtained from N_2_ adsorption isotherm in the relative pressure range of 0.05–0.30. ^b^ Total pore volume calculated as the amount of N_2_ adsorbed at *P*/*P*_0_ = 0.90. ^c^ Micropore volume calculated via *t*-plot method.

**Table 2:** Compositional analysis of H-SSZ-39, In/H-SSZ-39, and Cr-In/H-SSZ-39 samples.

Sample	In Content	Cr Content	Al Content	Si Content	Si/Al
	(wt%)	(wt%)	(wt%)	(wt%)	
H-SSZ-39	/	/	2.44	39.41	16
In/H-SSZ-39	5.5	/	2.45	40.33	16
Cr-In/H-SSZ-39	4.7	0.075	2.90	43.06	15

**Table 3:** Surface element compositions of In/H-SSZ-39 and Cr-In/H-SSZ-39 samples.

Sample	${\mathrm{InO}}^{+}/({\mathrm{InO}}^{+}+{\mathrm{In}}_{2}O_{3}+In{\left(OH\right)}_{3-z}^{z+})$	O_β_/(O_α_ + O_β_ + O_γ_)	Cr^3+^/(Cr^3+^ + Cr^6+^)
In/H-SSZ-39	0.49	0.36	/
Cr-In/H-SSZ-39	0.54	0.40	0.58
Used Cr-In/H-SSZ-39	0.54	0.40	0.57

**Table 4:** The strength and quantity of surface acid sites of In/H-SSZ-39 and Cr-In/H-SSZ-39 samples based on NH_3_-TPD measurements.

Sample	Peak I		Peak II		Peak III		*Q*_total_
	*T* (°C)	*Q* (mmol g^−1^)	*T* (°C)	*Q* (mmol g^−1^)	*T* (°C)	*Q* (mmol g^−1^)	(mmol g^−1^)
In/H-SSZ-39	178	1.098	477	0.186	546	0.529	1.813
Cr-In/H-SSZ-39	180	1.236	471	0.195	540	0.587	2.018
Used Cr-In/H-SSZ-39	182	1.145	470	0.184	538	0.552	1.881

## Data Availability

The original contributions presented in this study are included in the article. Further inquiries can be directed to the corresponding author.
